# Mavacamten in adolescent obstructive hypertrophic cardiomyopathy: SCOUTing a way forward

**DOI:** 10.1007/s10741-026-10655-x

**Published:** 2026-07-04

**Authors:** Joshua D. Bennetts, Doan T. M. Ngo, Baljash Cheema, Kaveh Hosseini, Nicolas Verheyen, Viktoria Santner, Daniela Tomasoni, Aaron L. Sverdlov

**Affiliations:** 1 College of Health, Medicine and Wellbeing, University of Newcastle, Newcastle, NSW Australia; 2 Hunter Medical Research Institute, New Lambton Heights, NSW Australia; 3 Division of Cardiology, Department of Medicine, Northwestern University Feinberg School of Medicine, Chicago, IL USA; 4 Department of Cardiology, Copenhagen University Hospital - Herlev and Gentofte, Copenhagen, Denmark; 5 Center for Translational Cardiology and Pragmatic Randomized Trials, Department of Biomedical Sciences, Faculty of Health and Medical Sciences, University of Copenhagen, Copenhagen, Denmark; 6 Division of Cardiology, Department of Internal Medicine, University Heart Center, Medical University of Graz, Graz, Austria; 7 Cardiology, ASST Spedali Civili di Brescia and Department of Medical and Surgical Specialties, Radiological Sciences, and Public Health, University of Brescia, Brescia, Italy; 8 Cardiovascular Department, John Hunter Hospital, Newcastle, NSW Australia

**Keywords:** Hypertrophic obstructive cardiomyopathy, Adolescents, Mavacamten, SCOUT-HCM

## Abstract

Paediatric obstructive hypertrophic cardiomyopathy (oHCM) is a rare inherited cardiac disease that may present from childhood through adolescence and is associated with substantial long-term morbidity and mortality. Its management is based largely on observational studies and clinical experience and currently focusses on symptom improvement through medical therapy and, when indicated, septal reduction therapy to alleviate left ventricular outflow tract (LVOT) obstruction. In adult oHCM, mavacamten has established cardiac myosin inhibition as an effective strategy to reduce LVOT obstruction, but paediatric trial evidence has been lacking. The SCOUT-HCM trial is the first randomised placebo-controlled trial in adolescents to investigate the efficacy and safety of mavacamten for symptomatic oHCM. Among 44 adolescents (mean age 14.7 ± 1.7 years) mavacamten significantly reduced Valsalva-provoked LVOT gradients at 28 weeks, confirming short-term physiological efficacy. Secondary and exploratory findings, including NYHA functional class, cardiac structure and biomarkers, showed a trend to improvement, without a signal of left ventricular systolic dysfunction. The positive results of SCOUT-HCM, align with larger and longer adult oHCM trials and are likely to influence clinical practice, supporting a cautious integration of mavacamten in adolescent oHCM management. Longer follow-up and broader paediatric studies should determine durability of benefit, validated patient-reported outcomes, exercise capacity, arrhythmia burden, long-term safety and the need for septal reduction therapy in paediatric oHCM.

## Introduction

Hypertrophic cardiomyopathy (HCM) is a common inherited cardiac disease typically characterised by asymmetrical left ventricular (LV) hypertrophy that cannot be explained by abnormal loading condition [1, 2]. Paediatric HCM exhibits considerable disease heterogeneity compared to adult populations and is associated with significant long-term morbidity and mortality, including life-threatening arrhythmias and sudden cardiac death [3–5]. Paediatric HCM includes sarcomeric disease driven by variants in contractile proteins, genotype-negative disease in which no pathogenic sarcomeric variant is identified, and HCM phenocopies such as metabolic, neuromuscular, mitochondrial and syndromic disorders [3, 6]. This distinction is particularly relevant when discussing sarcomere-targeted therapy such as mavacamten, because phenocopies have different biology, clinical course and treatment considerations. Importantly, paediatric HCM is not simply adult disease presenting earlier; it occurs in the context of growth, exercise participation, family-based decision-making, schooling, reproductive counselling and transition into adult inherited cardiac disease care [1–3, 6].

Currently, there are no approved disease-specific pharmacotherapies for paediatric obstructive HCM. Pharmacological interventions are largely empirical, derived from adult trials and clinical practice, and focus on symptomatic relief with beta-blockers, non-dihydropyridine calcium channel blockers, and disopyramide [1, 2, 7, 8]. These therapies can reduce symptoms and dynamic obstruction, but they do not directly target the sarcomeric hypercontractility that underpins many cases of obstructive HCM [8]. SCOUT-HCM (Study of Mavacamten in Adolescents with Symptomatic Obstructive Hypertrophic Cardiomyopathy) is therefore important not simply because it tests another adult therapy in adolescents, but because it is the first randomised paediatric trial of a treatment designed to target the hypercontractile sarcomere in symptomatic obstructive HCM addressing an unmet need [8, 9].

In adults, cardiac myosin inhibitors have shifted the therapeutic discussion from non-specific negative inotropy toward targeted sarcomere modulation. In adult obstructive HCM, mavacamten and aficamten have demonstrated substantial reductions in LV outflow tract (LVOT) gradients and improvements in symptoms, functional class, exercise capacity and health status, although effects on hard clinical outcomes and prognosis remain unproven [8, 10–12]. Experience in non-obstructive HCM has shown that target engagement, biomarker improvement and imaging changes do not necessarily translate into definitive benefits [13]. These developments have prompted reconsideration of the traditional beta-blocker-first paradigm in selected patients, although long-term safety, durability of benefit, treatment sequencing and patient selection remain unresolved. However, paediatric HCM is more heterogeneous and carries a much longer disease horizon [3, 4]. SCOUT-HCM therefore extends an established adult pharmacological effect into adolescents, while raising paediatric questions around growth, exercise and sports participation, adherence, reproductive counselling, family-based decision-making and transition to adult inherited cardiomyopathy care [9].

## SCOUT-HCM: trial design and key findings

To answer that question, SCOUT-HCM enrolled symptomatic adolescents aged 12 to < 18 years with obstructive HCM [9]. Other key inclusion criteria included a Valsalva-provoked LVOT gradient ≥ 30 mmHg, maximal LVOT gradient ≥ 50 mmHg at rest, during Valsalva or after exercise, left ventricular ejection fraction (LVEF) ≥ 60%, and New York Heart Association (NYHA) functional class II or III symptoms. This trial excluded patients with HCM phenocopies, such as Noonan syndrome and Fabry disease.

Patients were stratified by age and beta-blocker use and randomised 1:1 to mavacamten or placebo for a 28-week double-blind treatment period [9]. Mavacamten was started once daily according to body weight and titrated based on echocardiographic assessment of Valsalva LVOT and LVEF. The primary endpoint was the change in Valsalva-provoked LVOT gradient from baseline to week 28 [9]. Key secondary endpoints were change in resting and post-exercise LVOT gradients, maximal LV wall thickness, E/e’ ratio, and patient-reported Hypertrophic Cardiomyopathy Symptom Questionnaire (HCMSQ) Shortness-of-breath score, peak oxygen uptake, NYHA class and mitral regurgitation. Exploratory endpoints included NT-proBNP and high-sensitivity cardiac troponins [9]. Since SCOUT-HCM was built around a haemodynamic primary endpoint in a small trial; the symptom, exercise, imaging and biomarker measures help interpret the results, but should not carry the same weight as the primary endpoint.

In total, 65 patients were screened, and 44 patients underwent randomisation to mavacamten (23 patients) or placebo (21 patients) [9]. The trial period was completed by all, but 1 patient: one placebo-treated patient requested unblinding and underwent myectomy due to symptom persistence. Baseline demographics and clinical characteristics were largely similar between the two cohorts, including baseline LVOT gradients (78.4 ± 34.1 mmHg in the mavacamten group vs. 80.8 ± 47.4 mmHg in the placebo group). Slight imbalances were observed in body mass index, NT-proBNP and high-sensitivity cardiac troponin I, with higher median biomarker concentrations in the mavacamten group at baseline [9]. Most patients were already receiving background HCM therapy, including beta-blockers in 83% and 86% of the mavacamten and placebo groups, respectively. In the mavacamten group, 91% of patients received an initial dose of 5 mg mavacamten daily and demonstrated ≥ 80% dose adherence [9].

Treatment with Mavacamten resulted in significant improvements in Valsalva-provoked LVOT gradients compared to placebo at week 28 [9]. The least-squares mean change in Valsalva LVOT gradient was − 48.5 mmHg with mavacamten and − 0.5 mmHg with placebo, corresponding to a between-group difference of − 48.0 mmHg (95% CI, − 67.7 to − 28.3; *P* < 0.001). Descriptive secondary analyses supported the primary endpoint, including reductions in resting and post-exercise LVOT gradients, maximal LV wall thickness and E/e′ ratio, and higher proportions of patients with NYHA class and mitral regurgitation improvement [9]. However, the HCMSQ Shortness-of-Breath score showed only a small between-group difference. Exploratory biomarker analyses showed lower NT-proBNP and high-sensitivity troponin concentrations with mavacamten compared to placebo, supporting a possible reduction in myocardial stress and injury [9]. Similar adverse event rates were seen in mavacamten and placebo groups (18 patients [78%] vs. 17 patients [81%], respectively). Two patients in each group had serious adverse events. No deaths occurred and no patient had an LVEF reduction to < 50%.

The most robust finding from SCOUT-HCM is the reduction in Valsalva-provoked LVOT gradient, while the symptom and exercise findings require more careful interpretation. Despite the marked haemodynamic improvement, the between-group difference in HCMSQ Shortness-of-Breath score was small, and the peak VO₂ data were not definitive. This is perhaps not surprising in adolescents, where symptoms and exercise capacity are influenced by more than LVOT obstruction alone, including physical conditioning, anxiety, school and sports participation, placebo response, short follow-up, small numbers, and the limitations of symptom tools not developed specifically for paediatric oHCM. The changes in wall thickness, E/e′, NT-proBNP and high-sensitivity troponin are encouraging and biologically plausible but should be viewed as signals of reduced myocardial stress and possible early remodelling, rather than evidence that durable disease modification has been achieved.

## Interpreting SCOUT-HCM: from proof of mechanism to clinical relevance

**Fig. 1:**
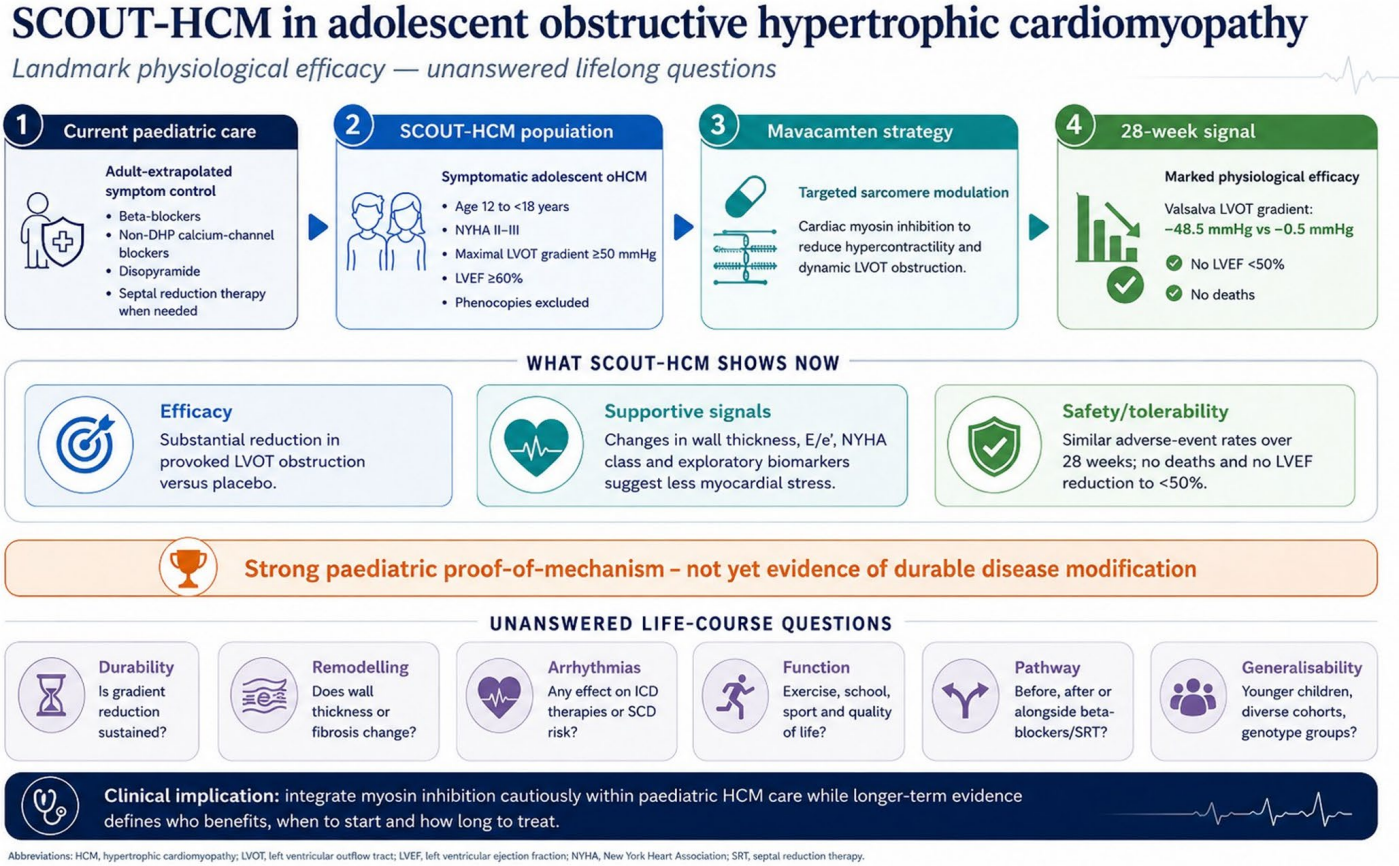
SCOUT-HCM in adolescent obstructive hypertrophic cardiomyopathy. SCOUT-HCM provides the first evidence that mavacamten can substantially reduce Valsalva-provoked LVOT obstruction in adolescents with symptomatic oHCM. This gradient reduction is the main short-term physiological finding of the trial. Changes in NYHA functional class, wall thickness, E/e′ and exploratory biomarkers act as supportive mechanistic signals, not as definitive evidence of disease modification. The modest HCMSQ Shortness-of-Breath improvement, non-definitive exercise-capacity findings and short 28-week follow-up highlight the need for longer-term studies assessing validated paediatric patient-reported outcomes, exercise capacity, arrhythmia burden, durability, safety and need for septal reduction therapy

The positive results of SCOUT-HCM align with larger and longer adult oHCM trials and are likely to influence clinical practice, supporting cautious integration of mavacamten into selected adolescent oHCM management. The significance of SCOUT-HCM lies in confirming that mavacamten lowers gradients in adolescent population, which was already anticipated from adult HCM studies [8], as well as in showing that the hypercontractile sarcomere is a modifiable therapeutic target in adolescents. This is an important shift for paediatric HCM, where treatment has historically been extrapolated from adult practice and centred on symptom relief. The reduction in LVOT obstruction was substantial, and the associated changes in wall thickness, E/e′ and biomarkers raise the possibility that reduction of LVOT obstruction by myosin inhibition may influence myocardial stress and early remodelling [9]. SCOUT-HCM should therefore be read as a strong paediatric proof-of-mechanism study, with longer follow up studies needed to assess the effect of mavacamten on the long-term natural history of childhood-onset HCM (Fig. 1).

Paediatric HCM is not simply childhood onset adult disease; it is associated with cumulative lifetime disease burden and poorer overall survival compared to adult populations [14]. The optimal timing of mavacamten initiation and length of treatment for paediatric populations is still unclear. It remains uncertain whether early intervention translates into sustained changes in disease progression and prognosis, rather than short-term physiological improvement alone. Robust data are needed to determine whether mavacamten improves outcomes beyond LVOT gradients, including remodelling, arrhythmia burden, exercise capacity, validated patient-reported outcomes and need for septal reduction therapy [4, 5, 9, 15]. These questions are particularly relevant in adolescence, when treatment decisions have to take into account growth, puberty, schooling, sport, evolving autonomy, family screening and transition to adult inherited cardiomyopathy care. Short-term haemodynamic endpoints cannot fully capture whether a treatment is practical, acceptable and meaningful across this longer life-course horizon.

## Treatment sequencing and generalisability

SCOUT-HCM also raises important questions about treatment sequencing. Surgical septal myectomy remains an option for adolescents with persistent symptoms despite optimal medical therapy or inability to tolerate medical therapy [16]. Whether mavacamten can replace or delay septal reduction therapy, was beyond the scope of SCOUT-HCM. Given that most participants were already receiving background HCM therapy, the current evidence most directly supports cautious integration or add-on use in selected symptomatic adolescents similar to those enrolled in SCOUT-HCM. Future clinical trials should define the role of mavacamten within the current treatment hierarchy: as add-on therapy, an alternative to conventional negative inotropes, a bridge to septal reduction therapy, or a strategy that may reduce the need for invasive intervention in selected patients.

The key strengths of SCOUT-HCM are its randomised, double-blind, placebo-controlled design, international multicentre enrolment, adolescent focus, echocardiography-guided dose titration and clinically relevant haemodynamic primary endpoint. There are several limitations of SCOUT-HCM that would temper immediate generalisation. The relatively small sample size precluded in-depth subgroup analyses, including assessment of sex differences. The population also remained predominantly White, did not include children younger than 12 years and excluded HCM phenocopies. Ongoing data collection for SCOUT-HCM should help address durability and longer-term safety in the enrolled population, but larger and more diverse paediatric clinical trials are required to provide definitive answers to these questions.

## Implementation in adolescent care

Mavacamten treatment requires structured dosing and monitoring schedules, including repeated echocardiographic assessment of LVOT gradient and LVEF to guide therapy and preserve systolic function [7]. In adolescents, this monitoring burden may interfere with school, sport, family routines and transition toward self-management, and would require careful integration into paediatric HCM care. The absence of LVEF reduction below 50% is reassuring, particularly in the context of weight-based dosing and echocardiography-guided titration, but should be interpreted cautiously given the small sample size and 28-week double-blind follow-up.

Paediatric HCM carries baseline risks of atrial and ventricular arrhythmias, ICD therapies, syncope and sudden cardiac death [5]. The impact of chronic myosin inhibition on arrhythmia burden in adolescents remains unknown. Longer-term follow-up is required to capture arrhythmia burden, syncope, ICD therapies and sudden death risk, rather than assuming that reducing LVOT obstruction will necessarily modify arrhythmic risk.

There are also several important drug-drug interactions to consider. Mavacamten is metabolised by CYP2C19 and, to a lesser extent, CYP3A4, and may interact with concomitant negative inotropes and other commonly used therapies. Non-dihydropyridine calcium channel blockers and hormonal oral contraceptives are other important drug classes with potential interactions [17, 18]. The latter are particularly important considerations for younger females who may require pharmacotherapeutic intervention into adulthood for obstructive HCM. As such, multidisciplinary management is advised that encompasses primary care, paediatric cardiologists, pharmacists and allied health.

## Future directions

SCOUT-HCM is an important first step towards optimising obstructive HCM therapy for paediatric populations. The next step is to move from proof-of-mechanism to clinical positioning. Longer follow-up from SCOUT-HCM will need to show whether gradient reduction is durable, systolic function remains stable, and favourable changes in biomarkers, wall thickness and filling indices translate into outcomes that matter to adolescents and families: validated patient-reported outcomes, exercise capacity, quality of life, arrhythmia burden, long-term safety and the need for septal reduction therapy. Broader studies are needed in younger children, more diverse populations and genotype-defined subgroups, with careful separation of sarcomeric HCM from syndromic or metabolic phenocopies. The practical question that will need an answer is where myosin inhibition belongs in the paediatric pathway: before, after or alongside beta-blockers, disopyramide and septal reduction therapy. Until longer-term data are available, SCOUT-HCM should be viewed as a landmark paediatric trial that establishes short-term efficacy and safety, supports cautious integration of mavacamten into selected adolescent oHCM care, and leaves open the larger question of whether early sarcomere modulation can alter the lifelong trajectory of paediatric HCM.

## Data Availability

No datasets were generated or analysed during the current study.
